# Indoor and outdoor malaria transmission in two ecological settings in rural Mali: implications for vector control

**DOI:** 10.1186/s12936-021-03650-0

**Published:** 2021-03-04

**Authors:** Moussa Keïta, Sidy Doumbia, Ibrahim Sissoko, Mahamoudou Touré, Sory Ibrahim Diawara, Drissa Konaté, Ambièlè Bernard Sodio, Sekou F. Traoré, Mahamadou Diakité, Seydou O. Doumbia, Nafomon Sogoba, Donald J. Krogstad, Jeffrey G. Shaffer, Mamadou B. Coulibaly

**Affiliations:** 1 West African International Center of Excellence for Malaria Research, Bamako, Mali; 2 Malaria Research and Training Center, Bamako, Mali; 3 Faculty of Medicine and Odonto Stomatology, University of Sciences, Techniques and Technologies of Bamako, Bamako, Mali; 4 Faculty of Science and Techniques, University of Sciences, Techniques and Technologies of Bamako, Bamako, Mali; 5 School of Public Health and Tropical Medicine, Tulane University, 1440 Canal Street, New Orleans, LA 70112 USA

**Keywords:** Malaria transmission, Outdoor, *An. gambiae* complex, Entomological Inoculation Rate (EIR), Pyrethrum spray catch, Human landing catch

## Abstract

**Background:**

Implementation and upscale of effective malaria vector control strategies necessitates understanding the multi-factorial aspects of transmission patterns. The primary aims of this study are to determine the vector composition, biting rates, trophic preference, and the overall importance of distinguishing outdoor *versus* indoor malaria transmission through a study at two communities in rural Mali.

**Methods:**

Mosquito collection was carried out between July 2012 and June 2016 at two rural Mali communities (Dangassa and Koïla Bamanan) using pyrethrum spray-catch and human landing catch approaches at both indoor and outdoor locations. Species of *Anopheles gambiae* complex were identified by polymerase chain reaction (PCR). Enzyme-Linked -Immuno-Sorbent Assay (ELISA) were used to determine the origin of mosquito blood meals and presence of *Plasmodium falciparum* sporozoite infections.

**Results:**

A total of 11,237 *An. gambiae* sensu lato (*s.l*.) were collected during the study period (5239 and 5998 from the Dangassa and Koïla Bamanan sites, respectively). Of the 679 identified by PCR in Dangassa, *Anopheles coluzzii* was the predominant species with 91.4% of the catch followed by *An. gambiae* (8.0%) and *Anopheles arabiensis* (0.6%). At the same time in Koïla Bamanan, of the 623 *An. gambiae s.l.*, *An. coluzzii* accounted for 99% of the catch, *An. arabiensis* 0.8% and *An. gambiae* 0.2%. Human Blood Index (HBI) measures were significantly higher in Dangassa (79.4%; 95% Bayesian credible interval (BCI) [77.4, 81.4]) than in Koïla Bamanan (15.9%; 95% BCI [14.7, 17.1]). The human biting rates were higher during the second half of the night at both sites. In Dangassa, the sporozoite rate was comparable between outdoor and indoor mosquito collections. For outdoor collections, the sporozoite positive rate was 3.6% (95% BCI [2.1–4.3]) and indoor collections were 3.1% (95% BCI [2.4–5.0]). In Koïla Bamanan, the sporozoite rate was higher indoors at 4.3% (95% BCI [2.7–6.3]) compared with outdoors at 2.4% (95% BCI [1.1–4.2]). In Dangassa, corrected entomological inoculation rates (cEIRs) using HBI were 13.74 [95% BCI 9.21–19.14] infective bites/person/month (ib/p/m) at indoor, and 18.66 [95% BCI 12.55–25.81] ib/p/m at outdoor. For Koïla Bamanan, cEIRs were 1.57 [95% BCI 2.34–2.72] ib/p/m and 0.94 [95% BCI 0.43–1.64] ib/p/m for indoor and outdoor, respectively. EIRs were significantly higher at the Dangassa site than the Koïla Bamanan site.

**Conclusion:**

The findings in this work may indicate the occurrence of active, outdoor residual malaria transmission is comparable to indoor transmission in some geographic settings. The high outdoor transmission patterns observed here highlight the need for additional strategies to combat outdoor malaria transmission to complement traditional indoor preventive approaches such as long-lasting insecticidal nets (LLINs) and indoor residual spraying (IRS) which typically focus on resting mosquitoes.

## Background

Malaria transmission is heterogeneous and heavily dependent on the local climatic and eco-geographical conditions. Variations are commonly observed at both the village and household levels [1, 2]. Vector control relies mainly on large-scale indoor residual spraying (IRS) and bed net distribution, both being recommended by the World Health Organization [3]. With proper implementation, these approaches have been shown to be highly effective in reducing human-mosquito contact and the burden of malaria [4–7]. In fact, IRS played a central role in the ultimate eradication of malaria in Europe in the 1950s [8], and recent elimination in some African countries [9]. Distribution of long-lasting insecticidal nets (LLINs) was a main driver for the decline in malaria incidence between 2000 and 2015, accounting for an estimated 68% of the 663 million clinical cases averted over this time period [5]. *Anopheles coluzzii*, *Anopheles gambiae* sensu stricto (*s.s*.) and *Anopheles arabiensis,* that belong to the *Anopheles gambiae* complex, and the *Anopheles funestus* group are the predominant malaria vectors in Mali. *Anopheles gambiae*+ and *An. coluzzii*, two of the four major malaria vectors in Africa, have been described as being mainly endophagic and endophilic [10–14]. There is increasing evidence of a decline in both endophagic and endophilic behaviour in the *An. gambiae s.s.* and *An. coluzzii* populations after the introduction of IRS and LLINs [15, 16]. Because they both target indoor biting and resting mosquitoes, their use over time and across space might have led to behavioural changes in vector populations [16–19]. The reaction of the mosquito populations to these indoor-use control strategies has jointly led to declining endophilic behavioural activity and increased outdoor biting rates. This phenomenon can be exacerbated in tropical areas where, due to high temperatures, human populations tend to remain outdoors for longer periods of time and are bitten before sheltering indoors. Therefore, it appears that current control strategies focusing on indoor-based methods may not be enough to eliminate malaria transmission in most endemic countries [20, 21]. Hence it is necessary to understand the importance, ecology and dynamics of outdoor transmission to develop and implement appropriate outdoor control methods. The objectives of this study were, therefore, to investigate potential changes in vector species composition, feeding behaviour and contribution to indoor and outdoor malaria transmission in two ecologically different situated in rural parts of Mali.

## Methods

### Study sites

**Fig. 1:**
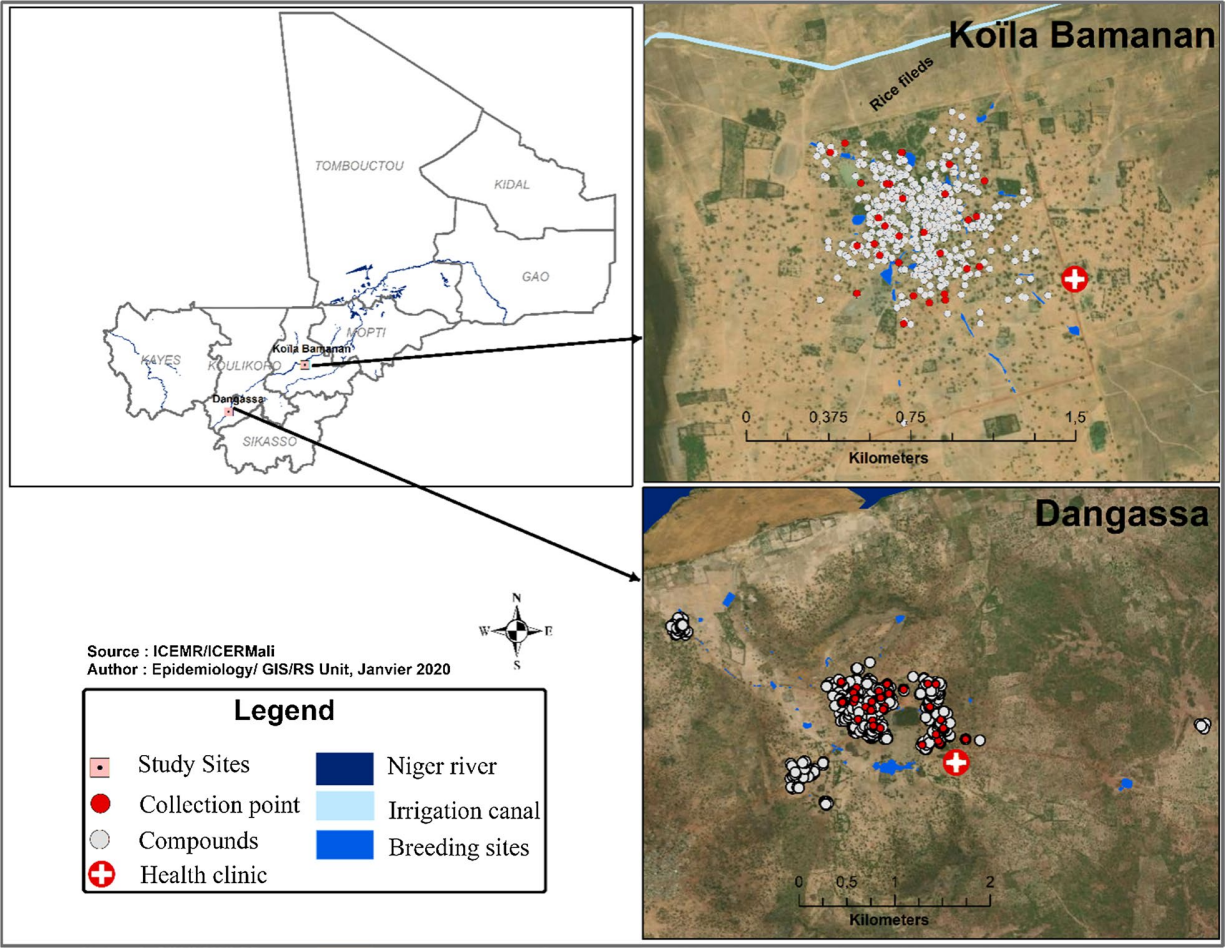
Map showing the study sites

Data was obtained from longitudinal mosquito population surveys conducted at two sites in Mali, the villages of Dangassa and Koïla Bamanan located in two distinct ecological settings (Fig. 1). At each study village, longitudinal surveys were carried out during 12-day field visits at the beginning (June-July), middle (August), the end (October) of the rainy season; and in the dry season (April) from 2012 to 2016.

Dangassa (8° 12′ 37.253″ W and 12° 8′ 46.279″ N) is located in the health district of Ouéléssébougou, 75 km away south of Bamako. Between the fringe of the village and the river Niger, lays a plain of about 1 km that floods in the rainy season. It is used for rice cultivation in the rainy season and vegetables gardening in the dry season. This is a Sudanian eco-geographical zone with a rainy season lasting from June to October and a dry season from November to May. The annual mean rainfall from 2012 to 2016 was 947.92 mm. The dominant winds are the monsoon (rainy season) and the harmattan (dry season).

Koïla Bamanan (5° 45′ 32.18″ W and 13° 38′ 24.026″ N) in the health district of Dioro, is located about 385 km south-east of Bamako. This is a Sahelian eco-geographical zone with a rainy season from July to October with about 250–500 mm of rain per year and a dry season from November to May. Koïla Bamaman is located in an irrigated area where controlled submersion rice cultivation is practiced. The dominant winds are the monsoon (rainy season) and the harmattan (dry season).

According to the 2012 Demographic and Health Survey (DHS) in Mali, the prevalence of malaria among children under 5 years old was 52% based on microscopy and 47% based on rapid diagnostic tests (RDTs) [22]. *Plasmodium falciparum* parasites accounted for 85–90% of malaria infections, while other parasite types included *Plasmodium malariae* (10–14%) and *Plasmodium ovale* (1%). *Anopheles gambiae* sensu lato (*s.l*.) (*An. coluzzii, An. gambiae, An. arabiensis*) and *An. funestus* group are currently the main vectors in Mali. In all the villages’ malaria control core intervention are chemoprevention by intermittent preventive treatment (IPT) in pregnant women and seasonal malaria chemoprevention (SMC) in children from 3 to 59 months, the use of long-lasting insecticidal nets (LLINs).

### Mosquito collection

Two collection methods were used for adult mosquitoes sampling: pyrethrum spray catch (PSC) and human landing catch (HLC). In total, 12 different surveys in each village over 5 years (2012–2016) were performed. During each survey, three rounds of HLC were set up with two collection points. Two volunteers (one indoor and one outdoor) collected mosquitoes at each of the two points making a total collection effort of 12 man-nights per survey. The collection sessions occurred from 18:00 h to 06:00 h indoors and outdoors in two randomly selected rooms. Collected mosquitoes were kept in separate cups, labelled by collection night, location and collection and time point. The volunteers rotated between indoor and outdoor positions after every hour to account for any biases due to variability in the attractiveness of individuals to mosquitoes [23, 24]. Volunteers were selected among young adult males at least 18 years old from the study site communities. The volunteers were trained prior to data collection periods and were informed about the study risks before agreeing to participate. Volunteers were offered treatment if they presented to health centers with malaria symptoms during the study. Informed consent was sought from room owners or users for the mosquito collections as the collections involved overnight stays. The collected specimens on human baits were morphologically identified [13, 14]. All these operations were supervised by two experienced entomologists.

During each survey, three sessions of PSC were conducted in the rooms to collect indoor resting mosquitoes from 07:00 h to 09:00 h according to the World Health Organization (WHO) standard protocol [25]. After each session, the collected mosquitoes were sorted according to their abdominal status (unfed, fed, half-gravid, and gravid) and dissected in two portions (head-thorax and abdomen) for the laboratory process. Each mosquito was kept in a labeled 1.5 ml eppendorf tube containing 80% ethanol. Samples were stored at − 20 °C freezer in the laboratory until used for further processing.

### Laboratory analysis of mosquitoes

The species composition of *An. gambiae* complex was determined by PCR [26]. The blood meal sources of freshly fed and half gravid *Anopheles* were analysed by a direct enzyme-linked immunosorbent assay (ELISA) using human antibodies [27]. Head and thorax portions of the preserved *Anopheles* specimens were tested for *P. falciparum* circumsporozoite proteins (CSPs) using the ELISA technique [28].

### Data management and analysis

Data were entered in StudyTRAX database management system (version v3.2.0802, StudyTRAX, Macon, GA). The Win BUGS application (version 1.4.1) was used to analyse the data. Pearson’s Chi square tests were used to compare proportions (species composition). The mosquito density, the human biting rates, the human blood index, the *P. falciparum* infection rates and the entomological inoculation rates were estimated using formulas in Table 1 [29]. Bayesian credible intervals (BCI) were also used to compare the proportions. All analyses were carried out with a 5% type I error threshold.

**Table 1:** Formulas used to calculate the entomological parameters

Entomological parameters	Formula
Densities of female mosquitoes per room	Total number of collected mosquitoes/total number of rooms
Human blood index	(Total number of positive in ELISA for human blood/total number of tested mosquitoes) × 100
Human biting rates (HLC)	Total number of collected mosquitoes/(total number of volunteers x number of rounds)
Human biting rates (PSC)	Total number of freshly fed mosquitoes/number of residents that have slept in the rooms the previous night
Sporozoite infection rate in mosquitoes	(Total positive in ELISA/total tested) × 100
Entomological Inoculation Rates (HLC and PSC)	HBR × (Total positive in ELISA/total tested)

### Ethics approval and consent to participate

This study was approved by the Institutional Review Board of Tulane University (11-255609) and the Ethics Committee of the Faculty of Medicine and Faculty of Pharmacy (FMPOS) at the University of Sciences, Techniques and Technologies of Bamako (USTTB) in Mali (2011/77/FMPOS). In each of the villages, community consent had been obtained prior to the occurrence of any study activities. Individual informed consent forms were obtained for PSCs and HLCs (for both room owners and data collectors) before starting mosquito collections.

## Results

### *Anopheles* mosquito species composition and abundance

During the study period, a total of 11,237 *An. gambiae s.l.* females were collected by PSC and HLC approaches. Among these specimens, 46.6% (5239/11,237) were collected in Dangassa and 53.4% (5998/11,237) in Koïla Bamanan. Based on logistics and practical considerations, a sub-sample was randomly selected across surveys to conduct molecular species identification. Of the 679 identified by PCR in Dangassa, *An. coluzzii* was the predominant species with a prevalence of 91.4% followed by *An. gambiae* (8.0%) and *An. arabiensis* (< 1%). In Koïla Bamanan, 623 *An. gambiae s.l.* were identified by the same methods and *An. coluzzii* represented 99%, *An. arabiensis* 0.8% and *An. gambiae* 0.2% of the catches. Overall, the *An. coluzzii* frequency was higher in Koïla Bamanan (99%) than in Dangassa (91.4%) (χ^2^ = 38.99, P < .001). No significant difference was observed between the frequencies of *An. arabiensis* (χ^2^ = 0.001, P = 0.970) and *An. gambiae* (χ^2^ = 0.081, P = 0.770) at both sites.

### Density and human blood index

**Fig. 2:**
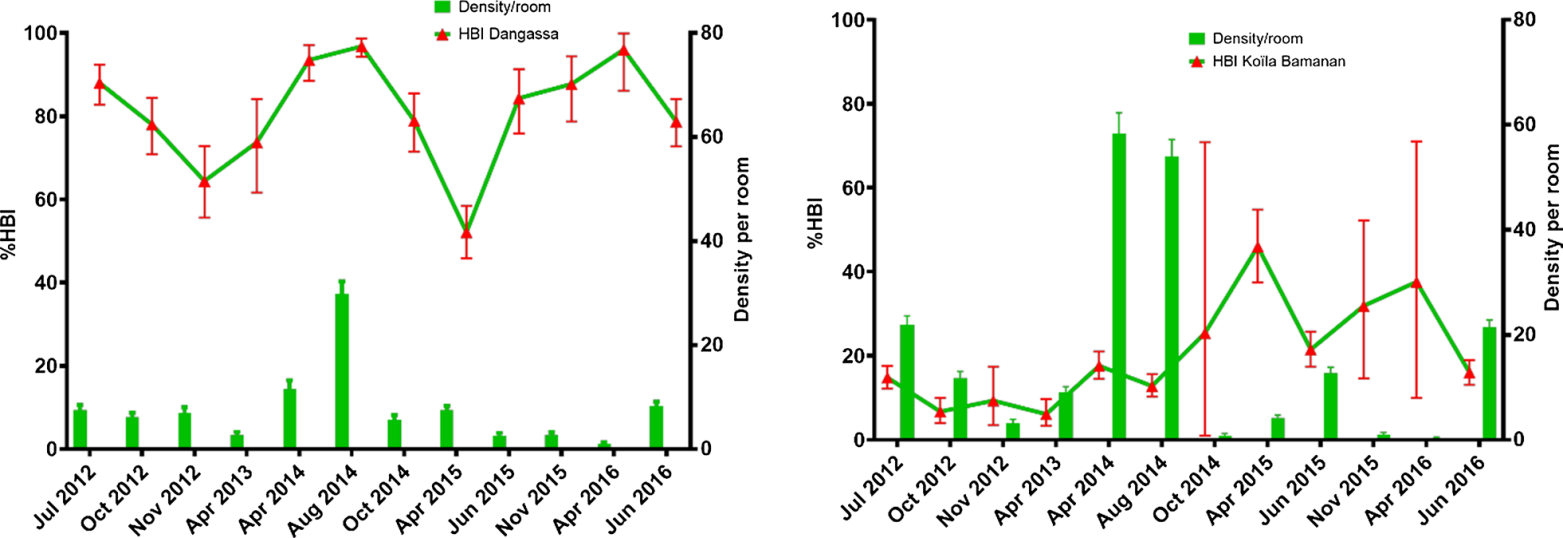
Monthly mean density (MMD) and human blood index (HBI) of *An. gambiae* s.l. in human dwellings in Dangassa & Koïla Bamanan from 2012 to 2016

In terms of abundance, Fig. 2 shows the monthly mean densities of *An. gambiae s.l.* in both localities using the PSC method. The mean density of *An. gambiae s.l.* over the entire study period was 12.8 [95% BCI 12.5–13.2] per room in Koïla Bamanan and 6.4 [95% BCI 6.2–6.7] per room in Dangassa. The highest densities were observed in April and August of 2014 for both villages. However, the densities of Koïla Bamanan were considerably higher than those of Dangassa. The lowest densities were observed in April 2016 for both study sites.

Over the 12 surveys, the mean HBIs of *An. gambiae s.l.* for Dangassa and Koïla Bamanan were 79.4% and 15.9%, respectively (Fig. 2). In Dangassa, the highest HBI was observed in August 2014 (96.8%) and the lowest HBI (52.1%) in April 2015, while in Koïla Bamanan, the lowest (6.7%) was observed in October 2012 and the highest (46.0%) in April 2015. HBIs were significantly higher in Dangassa than in Koïla Bamanan (χ2 = 1913.6, P < 0.001) during the entire study period.

### Monthly human biting rates (MHBRs), sporozoite infection rates (SIR) and entomological inoculation rates (EIRs)

**Fig. 3:**
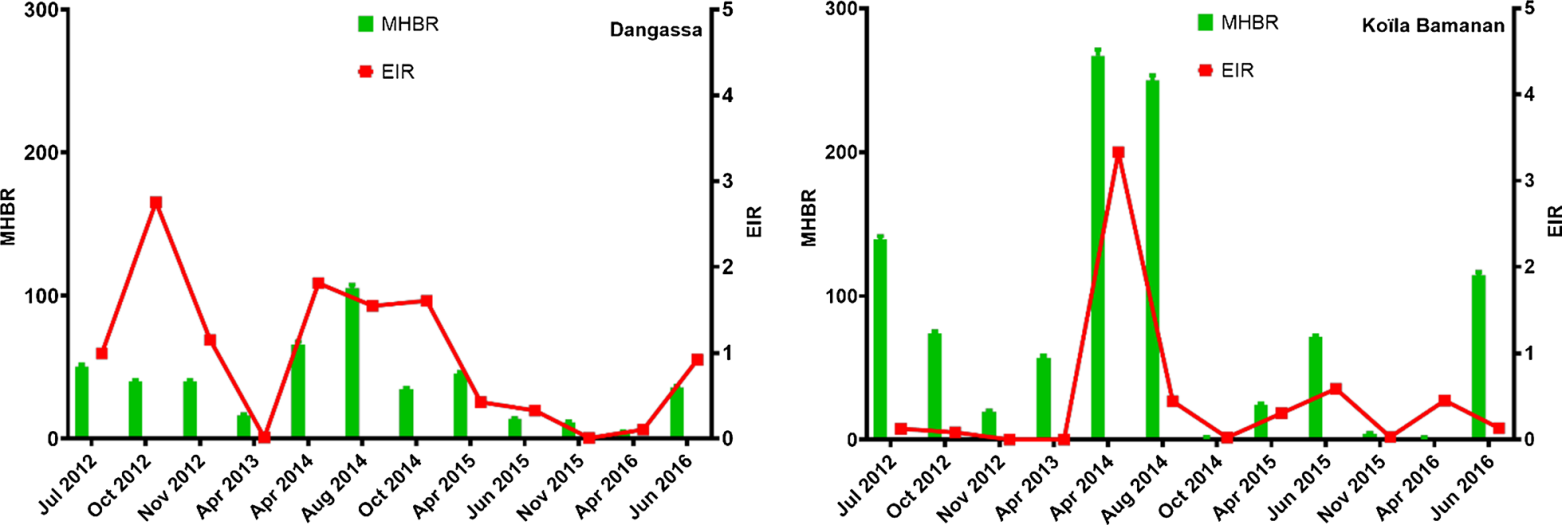
Monthly human biting rate (MHBR) and entomological inoculation rate (EIR) of *Anopheles gambiae* s.l. mosquitoes collected by spray-catch from Dangassa and Koïla Bamanan form July 2012 to June 2016

Figure 3 shows the human biting rates and the entomological inoculation rates from PSC in Dangassa and Koïla Bamanan respectively. The average monthly human biting rate (MHBR) was 2.3 times higher in Koïla Bamanan (69.8 bites/person/month) than in Dangassa (30 b/p/m). Monthly variations in MHBRs were observed at both sites. The highest MHBRs were recorded in April and August 2014. However, they were higher in Koïla Bamanan (266.6 and 249.6 b/p/m) than in Dangassa (65.9 and 105.5 b/p/m) (Fig. 3).

There was no significant difference in overall (over the entire study period) sporozoite infection rates (SIR) between the two study sites (infection rates were 2.9%, [95% BCI 2.3–3.6 for Dangassa versus 2.6% [95% BCI 2.2–3.1] for Koïla Bamanan; Table 2). Monthly and annual variations were observed at both sites. In Dangassa, the highest SIRs were observed in October (8.9%) in both 2012 and October 2014 (5.9%). The peaks of the infection rates were observed in October in both Dangassa Koïla Bamanan. In Koïla Bamanan, in addition to the peak in October (6.4% in 2014), there was a second peak that was observed in April (7.1% in 2014 and 78.5% in 2016).

**Table 2:** Sporozoite infection rate (SIR) and entomological inoculation rate (EIR) of *Anopheles gambiae* s.l. mosquitoes collected by spray-catch from Dangassa and Koïla Bamanan form July 2012 to June 2016

Survey	Dangassa					Koïla Bamanan				
	#Tested	IR (%)	BCI	EIR	BCI	#Tested	IR (%)	BCI	EIR	BCI
Jul 2012	226	2.2	[0.7–4.5]	0.99	[0.33–2.01]	656	0.6	[0.2–1.3]	0.13	[0.04–0.28]
Oct 2012	181	8.9	[5.2–13.4]	2.75	[1.61–4.17]	351	1.7	[0.6–3.3]	0.09	[0.03–0.16]
Nov 2012	224	4.5	[2.2–7.5]	1.15	[0.56–1.94]	96	0.1	[0.0–1.1]	0.00	[0.00–0.02]
Apr 2013	81	0.1	[0.0–1.3]	0.01	[0.00–0.15]	269	0.0	[0.0–0.4]	0.00	[0.00–0.01]
Apr 2014	171	2.9	[1.0–5.9]	1.81	[0.60–3.66]	875	7.1	[5.5–8.9]	3.33	[2.57–4.17]
Aug 2014	596	1.5	[0.7–2.6]	1.55	[0.72–2.69]	1078	1.4	[0.8–2.2]	0.45	[0.25–0.70]
Oct 2014	169	5.9	[2.9–9.9]	1.61	[0.79–2.71]	16	6.4	[0.2–22.1]	0.03	[0.00–0.09]
Apr 2015	337	1.8	[0.7–3.5]	0.43	[0.16–0.82]	183	2.8	[0.9–5.6]	0.31	[0.10–0.62]
Jun 2015	141	2.9	[0.8–6.2]	0.33	[0.09–0.72]	572	3.9	[2.4–5.6]	0.59	[0.37–0.86]
Nov 2015	121	0.1	[0.0–0.9]	0.01	[0.00–0.09]	47	2.2	[0.1–7.9]	0.03	[0.00–0.11]
Apr 2016	46	2.3	[0.1–8.0]	0.11	[0.00–0.36]	14	78.5	[54.5–94.9]	0.45	[0.31–0.58]
Jun 2016	369	3.3	[1.7–5.3]	0.92	[0.48–1.50]	963	0.7	[0.3–1.4]	0.13	[0.05–0.25]
Total	2662	2.9	[2.3–3.6]	0.70	[0.56–0.86]	5120	2.6	[2.2–3.1]	0.29	[0.24–0.34]

Given the fact that mosquitoes can feed on different hosts, to have a correct estimation of the EIR, we multiplied it by the proportion of mosquitoes which have taken their blood meal on human following the ELISA test. This correction is especially required when the human blood index is less 60%. The mean EIR was significantly higher in Dangassa (0.70 infective bites/person/month = ib/p/m) than in Koïla Bamanan (0.29 ib/p/m; Fig. 3). In Dangassa, the highest monthly EIRs were observed during the months of October 2012, April and October 2014, (2.75; 1.81 and 1.61 ib/p/m). In Koïla Bamanan, the highest EIRs were recorded for April 2014 and June 2015 (3.33 and 0.59 ib/p/m, respectively). As with the sporozoite infection rates, EIRs also were subject to monthly and annual variations.

### Outdoor vs indoor biting activities of *An. gambiae s.l.* in Dangassa and Koïla Bamanan

**Fig. 4:**
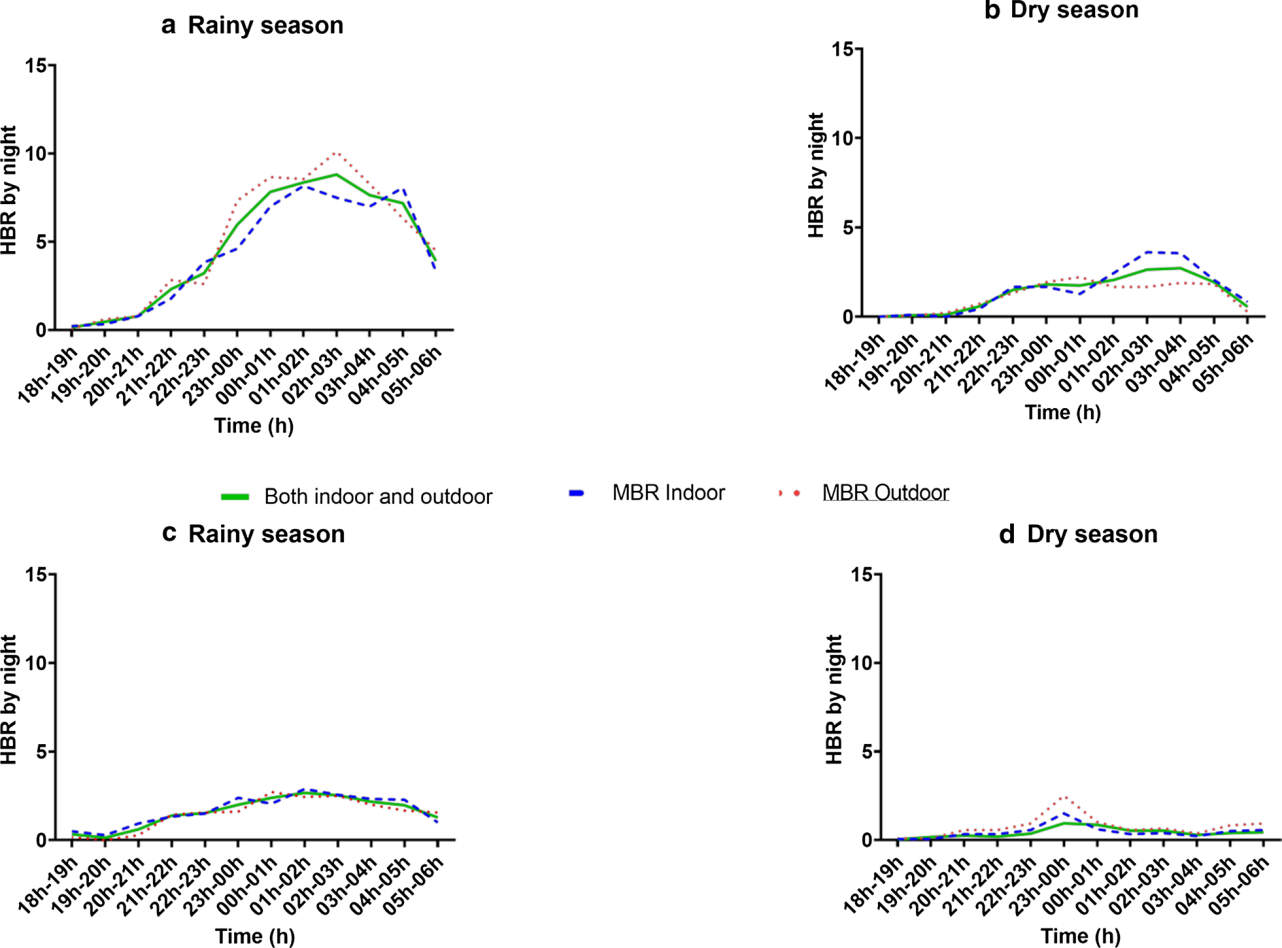
Hourly biting activity of *An. gambiae* s.l in Dangassa (**a** and **b**) and Koïla Bamanan (**c** and **d**) during study period by season. Rainy season covers June to October and dry season covers November to May

Data for comparing the location of biting activities (indoor versus outdoor locations) were generated using mosquitoes collected by human landing catches. Hourly variation of HBRs showed a wide patterns of variation for both indoor and outdoor locations in both villages (Fig. 4).

Figure 4 shows HBR per hour and by season. HBR was higher during the second part of the night indoors and outdoors regardless of site and season. A peak of HBR was observed outdoors between 02:00 h and 03:00 h, and then two peaks respectively between 01:00 h–02:00 h and 04:00 h–05:00 h during the rainy season at Dangassa (Fig. 4a). In the dry season, the peak was observed between 02:00 h and 04:00 h, but it was lower than in the wet season (Fig. 4b). At Koïla Bamanan, two peaks were observed indoor during the rainy season, where the first occurred between 23:00 h and 00:00 h, and the second occurred between 01:00 h and 02:00 h (Fig. 4c). In the dry season, a single peak was observed between 23:00 h and 00:00 h indoor and outdoor at Koïla Bamanan (Fig. 4d).

**Fig. 5:**
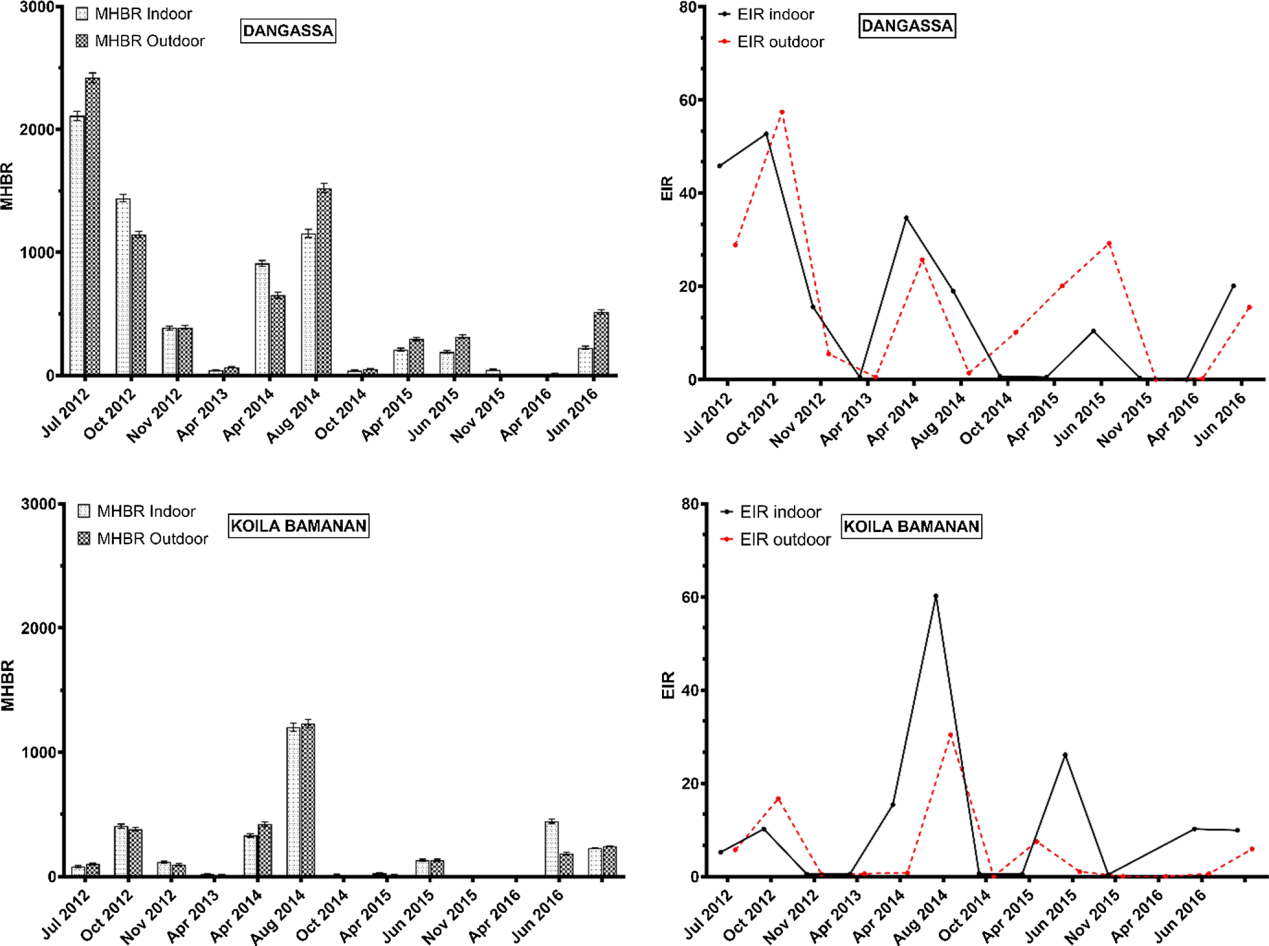
Monthly Human Biting Rate (MHBR) and Entomological Inoculation Rate (EIR) of *Anopheles gambiae* s.l. mosquitoes collected by Human Landing Catch (HLC) at Dangassa and Koïla Bamanan indoors and outdoors from July 2012 to June 2016

In Dangassa, the highest MHBRs were observed in July, October and November 2012, and in April and August 2014 (Fig. 5). The average HBR over the study period was significantly higher outdoors (648.9 b/p/m [BCI 642.6–655.2]) than indoors (560.3 b/p/m [BCI 554.7–565.9]) suggesting an exophagic coefficient of 1.2 (648.9/560.3).

In Koïla Bamanan, the highest MHBRs were observed in October 2012, April and August 2014 and in June 2016 indoor and outdoor (Fig. 5). As observed in Dangassa, in Koïla Bamanan the average HBR over the study period was significantly higher for outdoor locations (242.4 b/p/m [BCI 238.1–246.7]) than indoor locations (228.9 b/p/m [BCI 225.1–232.6]) with an exophagic coefficient of 1.1 (242.4/228.9). HBRs were significantly higher in Dangassa than in Koïla Bamanan, both indoors and outdoors.

In Dangassa, the sporozoite infection rate in *An. gambiae* s.l. was 3.1% [BCI 2.1–4.3] and 3.6% [BCI 2.4–5.0] for indoor and outdoor locations, respectively. No significant differences were observed between these two rates. The sporozoite infection rates were subject to wide month-to-month variability. In Koïla Bamanan, the average sporozoite infection rate was 4.3% [95% BCI 2.7–6.3] and 2.4% [95% BCI 1.1–4.2] for indoor and outdoor locations, respectively. The sporozoite infection rate indoors was 1.8 times higher than outdoors, though no significant difference was observed between the two. Similar monthly variations were observed both indoors and outdoors for Dangassa (Table 3).

**Table 3:** Sporozoite infection rate (SIR) of *Anopheles gambiae* s.l. mosquitoes collected by human landing catch indoors and outdoors in Dangassa and Koïla Bamanan from July 2012 to June 2016

Surveys	Dangassa				Koïla Bamanan			
	Indoor		Outdoor		Indoor		Outdoor	
	#Tested	IR (%) BCI	#Tested	IR (%) BCI	#Tested	IR (%) BCI	#Tested	IR (%) BCI
Jul 2012	232	2.2 [0.7–4.4]	88	1.2 [0.0–4.3]	16	6.5 [0.2–22.1]	18	5.7 [0.2–19.8]
Oct 2012	165	3.7 [1.4–7.0]	180	5.0 [2.3–8.6]	81	2.5 [0.3–6.8]	69	4.4 [0.9–10.3]
Nov 2012	75	4.0 [0.9–9.5]	74	1.4 [0.0–5.1]	22	0.5 [0.0–4.4]	18	0.6 [0.0–5.3]
Apr 2013	7	1.4 [0.0–13.0]	12	0.8 [0.0–7.8]	4	2.3 [0.0–22.1]	2	4.3 [0.0–41.9]
Apr 2014	158	3.8 [1.4–7.3]	77	3.9 [0.8–9.2]	65	4.7 [1.0–10.9]	56	0.2 [0.0–1.8]
Aug 2014	124	1.6 [0.2–4.5]	131	0.1 [0.0–0.8]	160	5.0 [2.2–8.9]	163	2.5 [0.7–5.3]
Oct 2014	5	1.8 [0.0–17.9]	10	20.1 [2.9–48.4]	2	4.3 [0.0–41.9]	0	0.0
Apr 2015	41	0.3 [0.0–2.4]	59	6.8 [1.9–14.4]	5	1.9 [0.0–18.0]	2	50.0 [2.7–97.2]
Jun 2015	37	5.5 [0.7–14.7]	65	9.3 [3.5–17.3]	20	20.1 [6.1–39.6]	13	0.8 [0.0–7.2]
Nov 2015	18	0.6 [0.0–5.3]	0	0	1	7.9 [0.0–72.7]	0	0.0
Apr 2016	2	4.3 [0.0–41.8]	6	1.6 [0.0–15.1]	0	0.0	0	0.0
Jun 2016	45	8.9 [2.6–18.7]	101	3.0 [0.6–7.1]	89	2.3 [0.3–6.2]	30	0.4 [0.0–3.3]
Total	909	3.1 [2.1–4.3]	803	3.6 [2.4–5.0]	465	4.3 [2.7–6.3]	371	2.4 [1.1–4.2]

EIRs showed monthly variations (Fig. 5). In Dangassa, the EIRs were estimated to be 13.74 [BCI 9.21–19.14] and 18.66 [BCI 12.55–25.81] ib/p/m, for indoor and outdoor locations, respectively. The EIR was 1.4 times higher outdoors than indoors, though the difference was not significant. The highest rates were observed in July and October 2012. In Koïla Bamanan, EIRs were 1.57 [BCI 2.34–2.72] and 0.94 [BCI 0.43–1.64] ib/p/m for indoor and outdoor locations, respectively. The highest rates were observed for August 2014 and June 2015. In contrast to Dangassa, the EIR was 1.7 times higher indoors than outdoors. However this difference was not significant.

## Discussion

Vector control remains a critical approach for malaria control and eliminations efforts. However, its success depends on periodic assessment of local malaria vector behaviour. This study analysed entomological parameters to better understand malaria transmission in two different ecological settings of Mali: Dangassa in the Sudan savanna region along the Niger River and Koïla Bamanan in the Sahelian region that conducts rice cropping. In both areas, *An. coluzzii* species was predominant. This may not reflect the whole species composition picture as the samples treated by PCR for species identification were: (1) collected before the event of universal LLINs coverage and (2) were not separated by collection method (i.e. HLC vs PSC). Mosquito species composition could be affected by vector control measures [30, 31] and could also vary according to the collection method. Previous studies conducted in Mali reported the predominance of this species in the Sudanese river savanna, flood prone and irrigated rice farming areas [32–35]. The authors explained their findings by the presence of favorable larval breeding sites to the development of this species such as ladder like brick pits, water puddles, ruts, flood prone plains and irrigated rice farming which were also common in our study sites. The highest density rates observed for Koïla Bamanan (twice) compared to Dangassa are most likely due to the rice cultivation which offers numerous suitable larval breeding habitats, especially during the earlier development stage of rice [36–38].

The mean densities were two times higher in Koïla Bamanan than in Dangassa. The density peaks are observed during the rainy season. However, another low amplitude peak other than that of the rainy season was observed in April in both localities. In Dangassa, the peak in the dry season could be explained by the availability of productive larval sites created in the river bed of River Niger when water recedes as reported by many studies across Africa [32, 34, 35, 39]. In Koïla Bamanan the dry season peak could be explained by vegetable cropping which creates suitable water puddles for larval development [40].

The outdoor human biting rates (HBR) were significantly higher than indoor biting rates. This could be due to the side effect of ongoing control measures like LLINs in these places as *An. gambiae* s.l. were known to be more endophagic than exophagic [41]. Indeed, several studies have shown changing its biting and resting behaviour from indoors to outdoors when LLINs were widely used in an area. Such an observation was also made in many African countries [35, 42–45]. However, recent data from Benin reported higher HBRs indoors than outdoors [41].

The human blood index (HBI) was significantly higher in Dangassa than in the irrigated rice farming area of Koïla Bamanan. This observation has now become common in Mali as rice farming has been associated with lower HBI as demonstrated by many studies [36, 46]. The same observation has been reported in Senegal [47]. Explanation of this situation has been attributed to personal protection practices as the highest densities of *An. gambiae s.l.* are known to occur in rice growing areas. Thus, mosquitoes are diverged to alternative hosts present in the area such as bovines, sheep, and goats as it is in Koïla Bamanan (unpublished observations by our study team). In addition, from 2008 to 2014, Koïla Bamanan was part of the Millennium Villages’ Project where LLINs were freely distributed and replaced every 3 years. These practices significantly improved ownership and usage of LLINs which may have led to a behavioural change in trophic preference in the malaria vector. Many studies across Africa showed that anthropophagic and endophilic individuals could become zoophagic and exophilic from the intensification in the use of LLINs [16, 17, 31, 48, 49].

This study revealed a higher indoor than outdoor EIR at Koïla Bamanan. The inverse relationship was observed in Dangassa where outdoor EIR was higher than indoor EIR. These findings may be due to the higher outdoor HBR observed in Dangassa than in Koïla Bamanan. The higher outdoor HBR and EIR than indoors in Dangassa could be explained by prior findings that *An. gambiae s.l.* is known to be anthropophagic and endophilic, but is becoming more exophilic and humans are staying outside houses longer and are bitten before going under mosquito nets. Other environmental factors, especially elevated ambient temperature, cause people to sleep with no protection against mosquito bites [41] or outdoors. In fact, several studies reported a high outdoor transmission rate [35, 42, 50]. This finding could be attributed to the use of LLINS distributed during prenatal screenings and mothers having completed the vaccination cycles of their babies who receive treated mosquito nets for free (since 2008). Also, the universal coverage in LLINS from May 2015 could itself cause mosquito behavioural changes.

## Conclusion

*Anopheles gambiae s.l.* was more anthropophilic in Dangassa than in Koïla Bamanan. Both sites had comparable EIR outdoors and comparable indoor transmission which was also high at both sites. These findings have important implications for the epidemiology and strategies for control of malaria in the study area. Additional control strategies are needed to complement ongoing interventions to better address the issue of outdoor transmission and reduce indoor and outdoor resting vectors using the tool package offered by integrated vector management (IVM).

## Data Availability

The data used and/or analysed in this study are available from the corresponding author on reasonable request.
